# Genome-wide characterization of the chitinase gene family in wild apple (*Malus sieversii*) and domesticated apple (*Malus domestica*) reveals its role in resistance to *Valsa mali*

**DOI:** 10.3389/fpls.2022.1007936

**Published:** 2022-11-07

**Authors:** Yakupjan Haxim, Gulnaz Kahar, Xuechun Zhang, Yu Si, Abdul Waheed, Xiaojie Liu, Xuejing Wen, Xiaoshuang Li, Daoyuan Zhang

**Affiliations:** ^1^ State Key Laboratory of Desert and Oasis Ecology, Xinjiang Institute of Ecology and Geography, Chinese Academy of Sciences, Ürümqi, China; ^2^ Xinjiang Key Laboratory of Conservation and Utilization of Plant Gene Resources, Xinjiang Institute of Ecology and Geography, Chinese Academy of Sciences, Ürümqi, China; ^3^ Turpan Eremophytes Botanical Garden, Chinese Academy of Sciences, Turpan, China; ^4^ University of Chinese Academy of Sciences, College of Resources and Environment, Beijing, China; ^5^ School of Life Sciences, Xinjiang Normal University, Ürümqi, China

**Keywords:** apple canker, chitinase, *Malus sieversii*, gene family, *Valsa mali*

## Abstract

Chitinases are responsible for catalyzing the hydrolysis of chitin and contribute to plant defense against fungal pathogens by degrading fungal chitin. In this study, genome-wide identification of the chitinase gene family of wild apple (*Malus sieversii*) and domesticated apple (*Malus domestica*) was conducted, and the expression profile was analyzed in response to *Valsa mali* infection. A total of 36 and 47 chitinase genes belonging to the glycosyl hydrolase 18 (GH18) and 19 (GH19) families were identified in the genomes of *M. sieversii* and *M. domestica*, respectively. These genes were classified into five classes based on their phylogenetic relationships and conserved catalytic domains. The genes were randomly distributed on the chromosomes and exhibited expansion by tandem and segmental duplication. Eight of the 36 *MsChi* genes and 17 of the 47 *MdChi* genes were differentially expressed in response to *V. mali* inoculation. In particular, *MsChi35* and its ortholog *MdChi41*, a class IV chitinase, were constitutively expressed at high levels in *M. sieversii* and domesticated apple, respectively, and may play a crucial role in the defense response against *V. mali*. These results improve knowledge of the chitinase gene family in apple species and provide a foundation for further studies of fungal disease prevention in apple.

## Introduction

*Malus sieversii* is the primary progenitor of many cultivars of domesticated apple (*M.* domestica) and is mainly distributed in Central Asia and Xinjiang, China (Volk et al., 2013; Duan et al., 2017). A previous study of the phylogenetic relationships of cultivated apple and its ancestor revealed that *M. sieversii* from Xinjiang, China, was most closely related to the *M.* domestica cultivar ‘Golden Delicious’ (Zhou and Li, 2000). *Malus sieversii* is an important species in natural forest of the Tianshan Mountains in Xinjiang, and features high genetic diversity and disease resistance, thus providing a valuable genetic resource for molecular breeding of cultivated apple (Chen et al., 2007; Zhang et al., 2007; Ballester et al., 2017). The fruit of *M. sieversii* contain higher phenolic and flavonoid contents compared with those of cultivated apple, and potentially could be used to breed cultivars that produce fruit with red flesh and high flavonoid content (Zhang et al., 2008).

Recent studies have reported that the wild *M. sieversii* population in the Tianshan Mountains has experienced a dramatic decline partly as a result of Valsa canker (Bozorov et al., 2019; Liu et al., 2020). Valsa canker is caused by the necrotrophic pathogen *Valsa mali*, which is a major threat to apple production in China, Japan, Korea, and Central Asia, where it leads to severe yield losses (Lee et al., 2006; Suzaki, 2008; Yin et al., 2015; Wang et al., 2016). Fungicide applications are not always effective to control fungal mycelia within the host xylem (Yin et al., 2016). The mining of candidate genes and development of disease-resistant cultivars are effective and practical approaches to control the disease. The transcriptomic changes in response to *V. mali* infection have been investigated previously in *M. sieversii* (Liu et al., 2021) and *M.* domestica (Yin et al., 2016). The plant–pathogen interaction, plant hormone signal transduction, flavonoid biosynthesis, and phenylpropanoid biosynthesis pathways of *M. sieversii* (Haxim et al., 2021; Liu et al., 2021), and chitin signaling pathway of *M.* domestica (Yin et al., 2016) were significantly enriched in response to infection by *V. mali.* Exploration of the disease-resistance genes in apple responsive to *V. mali* infection is important for disease prevention in domesticated and wild apple species, especially *M. sieversii*.

Chitinases (EC 3.2.1.14) are glycosyl hydrolases that breakdown glycosidic bonds in chitin, a major structural component of fungal cell walls, and play important roles in plant defense responses (Grover Plant Chitinases, 2012; Hamid et al., 2013). Plant chitinase hydrolyzes the β-1,4-glycosidic bonds of chitin into chitin oligosaccharides during fungal infection and these oligosaccharides activate the immune responses of the host plant (Shibuya and Minami, 2001; Kaku et al., 2006; Hamid et al., 2013). To date, more than 175 different families of glycosyl hydrolases have been identified based on sequence similarity (http://www.cazy.org). On the basis of catalytic domains and amino acid sequence similarity, chitinases have been classified into the glycosyl hydrolase 18 (GH18) and 19 (GH19) families (Henrissat and Bairoch, 1993; Hamid et al., 2013). In addition, to reflect phylogenetic relationships, plant chitinases have been categorized into five classes (Class I to V), of which the GH18 family comprises classes III and V, and the GH19 family contains classes I, II, and IV (Grover Plant Chitinases, 2012). Chitinases are involved in the response to diverse abiotic stresses, such as heat, cold, salt, wounding, drought, heavy metal toxicity, ozone, and ultraviolet light, as well as a variety of plant phytohormones, including jasmonic acid, salicylic acid, ethylene, cytokinin, and indole acetic acid (Kasprzewska, 2003; Su et al., 2014). Members of the chitinase family from plant species are able to inhibit fungal growth *in vitro* (Li et al., 2019) and *in vivo* (Xiao et al., 2007), and therefore are potential candidates with which to enhance plant resistance to fungal pathogens (Kumar et al., 2018).

The availability of plant genomic sequence data allows genome-wide mining of the chitinase gene family. Recently, the chitinase gene family has been identified in the genomes of several tree species, such as *Populus trichocarpa* (Zhang et al., 2022), *Eucalyptus grandis* (Tobias et al., 2017), and *Hevea brasiliensis* (Misra, 2015). However, to date, the chitinase genes of apple species have not been systematically analyzed. The availability of whole-genome sequences for apple species, including *M. sieversii* and *M.* domestica, enables genome-wide identification of chitinase genes. In this study, the chitinase family members in the genomes of *M. sieversii* and *M. domestica* were surveyed and their phylogenetic relationships, gene structure, and gene duplication events were analyzed. In addition, the expression patterns of the chitinase genes in response to *V. mali* infection were analyzed. The present results extend knowledge of chitinases in apple species and might provide effective gene resources for improvement of apple resistance to *V. mali*.

## Materials and methods

### Plant material and pathogen infection

*Malus sieversii* (Ledeb.) M.Roem. seeds were purchased from the Nature and Wildlife Conservation Station of Xinyuan County, Xinjiang, China. *Malus* domestica ‘Golden Delicious’ seeds were purchased from the Liqun Nursery Co. Ltd. (Shandong, China). The seeds were germinated in a petri dish after removal of the husks. Three-week-old seedlings were planted in pots containing a mixture of soil and vermiculite and were grown at 24°C under a 16 h/8 h (light/dark) photoperiod. The *V. mali* isolate EGI1 (Liu et al., 2020) was grown on potato dextrose agar (PDA) medium (Rishui BioTechnologies, Qingdao, China) at 25°C for 3 days. Six-month-old plants were infected with *V. mali* as described previously (Liu et al., 2020; Haxim et al., 2021). Briefly, mycelial plugs (diameter: 5 mm) were excised from 3-day-old cultures of isolate EGI1 on PDA medium. Detached leaves of the host plant were wounded with a fabric pattern wheel and then inoculated with the mycelial plugs. The inoculated leaves were placed in petri dishes sealed with parafilm and were incubated in the dark at 25°C.

### RNA isolation and real-time quantitative PCR analysis

Total RNA was isolated from *M. sieversii* leaves collected at different time points (0, 1, 2, and 5 days post-inoculation [dpi]) after inoculation with *V. mali* using the E.Z.N.A.^®^ Plant RNA Kit (No. R6827, Omega Bio-tek, Norcross, GA, USA) in accordance with the manufacturer’s instructions. The first-strand cDNA was synthesized from 1 µg total RNA using the PrimeScript RT reagent Kit with gDNA Eraser (No. RR047Q, Takara, Dalian, China). To validate the expression level of the chitinase genes, gene-specific primers (**Table S1**) were designed using DNAMAN version 9.0 software (Lynnon BioSoft, Vaudreuil, Quebec, Canada) and synthesized by Sangon Biotech (Shanghai, China). The transcripts of the target genes were detected using the TB Green Premix Ex Taq II Kit (No. RR820A, Takara) with the CFX96 Real-Time PCR Detection System (Bio-Rad, Hercules, CA, USA). The thermal profile for qRT-PCR was as follows: preheating at 95°C for 30 s, then 40 cycles of 95°C for 5 s and 58°C for 30 s. A melting curve analysis was conducted to confirm the amplification specificity. The relative transcript abundance of the chitinase genes was analyzed with the 2^−ΔΔ^ *^C^* ^t^ method (Livak and Schmittgen, 2001). The *EF-1α* (Elongation factor 1-α) gene has been evaluated for internal reference in apple species (Zhu et al., 2019) and served as reference in our previous work (Liu et al., 2021; Haxim et al., 2021; Liu et al., 2021) because of its stable expression in our previous work under *V.mali* infection. Therefore, the EF-1α gene was also used as an internal reference for the qPCR quantification in the present study. Each sample comprised three biological replicates and each biological replicate was analyzed with three technical replicates. Statistical analysis of the data was performed with analysis of variance using SPSS 18 software (SPSS, Chicago, IL, USA).

### RNA-sequencing data analysis

Transcriptome data for *M. sieversii* (NCBI BioProject: PRJNA687214) (Liu et al., 2021) and *M.* domestica (NCBI SRA accession: SRP034726) (Yin et al., 2016) in response to *V. mali* infection were used to analyze differentially expressed genes. The fragments per kilobase of transcript per million fragments mapped (FPKM) value was calculated for each gene. Differential expression analysis of three biological replicates per condition was performed using the ‘DESeq’ R package (1.18.0). The *P*-values were adjusted using the Benjamini–Hochberg approach to control the false discovery rate. Transcripts with an adjusted *P-*value < 0.05 and log_2_ fold change > 1 were considered to be differentially expressed. The log_2_ fold-change values were used for heatmap generation.

### Genome-wide identification of chitinase genes

To identify potential chitinase genes in the two apple species, the *M. sieversii* genome (version JAHTLV010000000) and *M.* domestica genome (Daccord et al., 2017) (GDDH13 version 1.1) were retrieved from the NCBI (https://www.ncbi.nlm.nih.gov/) and GDR (https://www.rosaceae.org/) databases, respectively. A hidden Markov model seed profile of Glyco_hydro_18 (PF00704) and Glyco_hydro_19 (PF00182) was downloaded from the Pfam database (Mistry et al., 2021) and chitinase genes in the genomes were identified using TBtools software (Chen et al., 2020). The SMART (Letunic et al., 2021) database was used to confirm the presence of chitinase domains with a cut-off E-value < 0.0001.

### Phylogenetic relationships, gene organization, and conserved motif analysis

To study evolutionary relationships, the full-length amino acid sequences of chitinase proteins from *M.* domestica, *M. sieversii*, and *Arabidopsis thaliana* were aligned using Clustal X2 (http://www.clustal.org/). A phylogenetic tree was generated using MEGA-X software (https://www.megasoftware.net/) with the maximum likelihood method and topological support was assessed by means of a bootstrap analysis with 1000 replicates. The exon–intron organization of the chitinase genes was visualized using TBtools (Chen et al., 2020). Conserved motifs and domains were identified with the MEME Suite (Bailey et al., 2006) and SMART database (Letunic et al., 2021), respectively, and were visualized using TBtools (Chen et al., 2020). The domain signatures were identified using the PROSITE database (Sigrist et al., 2013). The SignalP 5.0 online server (Almagro Armenteros et al., 2019) was used to identify signal peptides in the chitinase proteins of *M. sieversii* and *M. domestica*.

### Chromosomal location and gene duplication analysis

The chromosomal location of the chitinase genes was visualized with the MG2C online tool (http://mg2c.iask.in/mg2c_v2.1/) based on genomic annotation data. Gene duplication events and synteny relationships of the chitinase genes were analyzed using the multiple collinearity scan toolkit MCScanX (Wang et al., 2012) and were visualized with TBtools (Chen et al., 2020). For tandem duplication events, two or more chitinase genes separated by five or fewer genes within an interval of 100 kb on the same chromosome were considered to be tandem duplicated genes (Wang et al., 2010; Zhao et al., 2018). Substitution rates of synonymous (*K*_s_) and non-synonymous (*K*_a_) chitinase genes were calculated with KaKs_Calculator 3.0 (Zhang, 2022). The evolutionary duplication time (*T*) was calculated with the formula *T* = *K*_s_/2λ × 10^−6^ million years ago (Mya), where λ = 1.5 × 10^−8^ in apple (Zhang et al., 2018).

### Prediction of *cis*-acting elements in promoter regions

The promoter sequence (the 2.0 kb DNA sequence upstream of the start codon) of each chitinase gene was retrieved from the *M. sieversii* genome (version JAHTLV010000000) and *M.* × *domestica* genome (GDDH13 version 1.1). The *cis*-acting elements in each gene was predicted using the PlantCARE online tools (Lescot, 2002).

### Gene co-expression network analysis for *M. sieversii*

Gene co-expression network analysis was performed using the weighted gene co-expression network analysis (WGCNA) tools from the BMKCloud platform (https://international.biocloud.net/zh/software/tools/list). Normalized gene expression data (FPKM > 1) in the 12 samples from *M. sieversii* were used as the input, and the expression level of the chitinase genes was used as a trait. The WGCNA modules of eigengenes correlated with chitinase gene expression profiles were identified with the default settings using a dynamic tree cut-off algorithm (minimum cluster size 30 and merging threshold function 0.3). Module membership (MM) was calculated based on Pearson correlation analysis between the expression level and the module eigengenes (ME; representative of the gene expression profile in each module). Gene significance (GS) was calculated as the correlation between the trait data and MEs.

### Transient expression assay

For the transient expression, the full length of *MsChi35* gene was cloned into pBI121 plant expression vector by using an In-fusion HD cloning kit (TAKRA, Code No.639648). The pBI121-MsChi35 was transiently expressed in detached *M.sieverssi* leaves by agroinfiltration method as described (Liu et al., 2021). After the 3 days of agroinfiltration, these leaves were wounded by a fabric pattern wheel and inoculated with *V.mali* isolate EGI1 as described (2.1) (Liu et al., 2020; Liu et al., 2021). Leaves inoculated with sterile ddH_2_O were used as controls. After a 4-days of inoculation of the *V. mali*, the lesion areas were measured by ImageJ software and lesion ratios (%) was calculated as proportion of lesion area in the whole leaf. Each sample contained ten leaves with three biological replicates. Statistical analysis of the data was performed with analysis of variance using SPSS 18 software (SPSS, Chicago, IL, USA).

## Results

### Genome-wide identification of chitinase gene family in two apple species

The whole-genome sequences of *M. sieversii* and *M.* × *domestica* were used for genome-wide exploration and phylogenetic analysis of the chitinase gene family. We used the Pfam database (Mistry et al., 2021) and the Glyco_hydro_18 (PF00704) and Glyco_hydro_19 (PF00182) domains to search for chitinases in the two apple genomes. The SMART (Letunic et al., 2021) database was used to verify the predicted genes. We accurately identified 36 putative chitinase genes in *M. sieversii*, of which 25 genes were members of the GH18 family and 11 genes belonged to the GH19 family (**Table S2**). In the *M.* × *domestica* genome, 47 chitinase genes were identified (**Table S2**), of which 33 genes were identified as members of the GH18 family and 14 genes belonged to the GH19 subfamily. For *M. sieversii*, the chitinase genes were annotated as *MsChi1* to *MsChi36*, whereas for *M.* × *domestica* the genes were annotated *MdChi1* to *MdChi47*, where the genes were numbered sequentially based on their chromosomal location in the genome.

The length of the 36 predicted MsChi proteins ranged from 101 (MsChi36) to 517 (MsChi1) amino acid residues, of which only MsChi1 was longer than 500 amino acids. The predicted molecular weight (MW) and isoelectric point (PI) ranged from 10.97 kDa (MsChi36) to 57.42 kDa (MsChi1) and from 4.34 (MsChi7) to 9.64 (MsChi23), respectively. Of the 36 MsChi proteins, 25 were predicted to contain a signal peptide in the C-terminus.

### Chromosomal location and phylogenetic analyses

**Figure 1:**
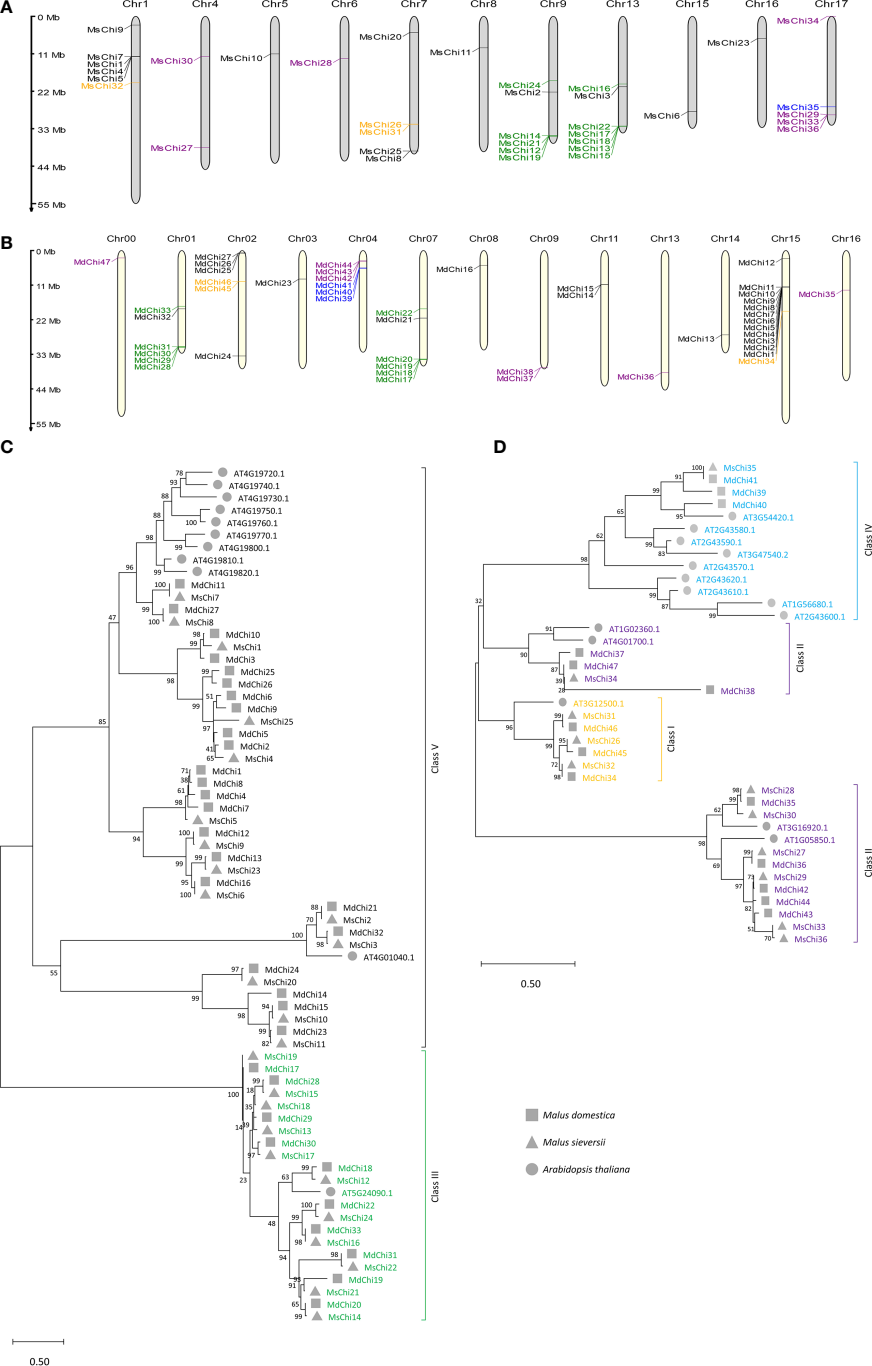
Chromosomal locations and phylogenetic relationship of *MsChis* and *MdChis*. Distribution of chitinase genes on *M.sieversii* **(A)** and *M.domestica* **(B)** chromosomes. MG2C online tools were used (http://mg2c.iask.in/mg2c_v2.1/) to depict chromosomal location, and genes were marked with short lines. The tandem duplicated gene pairs are marked by red braces. The major classes are represented as follows: orange=Class I, purple=Class II, green=Class III, blue= Class IV and black=Class V. Scale bar represents Mb. The full-length of amino acid sequences of putative chitinase protein from two glycosyl hydrolase families: GH18 **(C)** Class III (green) and V (black), and GH19 **(D)** Class IV (sky blue), Class II (purple), Class I (orange) in *M.domestica*, *M.sieversii* and *A.thaliana* were aligned using ClustalW method in MEGAX. An unrooted phylogenetic tree was built using the Maximum Likelihood method with 1000 replicates. The roman numerals (I–V) representing each gene cluster and genes from each species were labelled with different shapes. The numbers at the nodes represent statistical frequency.

To accurately locate the chromosomal position of the genes, a chromosomal distribution map was constructed based on the start–end position of each chitinase gene. The 36 *MsChi* genes were mapped to 11 chromosomes, whereas the 47 *MdChi* genes were distributed on 13 chromosomes (**Figures 1A, B**).

To explore the evolutionary relationships of the chitinase gene family, an unrooted phylogenetic tree for the 36, 47, and 25 chitinase proteins from *M. sieversii*, *M.* × *domestica*, and *A. thaliana*, respectively, was constructed using the maximum likelihood method. The GH18 subfamily was grouped into three lineages, whereas the GH19 subfamily was grouped into four branches. Based on the classification of *A. thaliana* chitinases (Passarinho and Vries, 2002), the *MsChi* and *MdChi* genes were further classified into five classes (**Figures 1C, D**). The number of members in each class differed between *M. sieversii* and *M.* × *domestica* (**Table S3**), especially in Class V. Interestingly, Class IV chitinases comprised only one member in *M. sieversii*, but three members in *M.* × *domestica*.

### Gene duplication and synteny analysis

**Figure 2:**
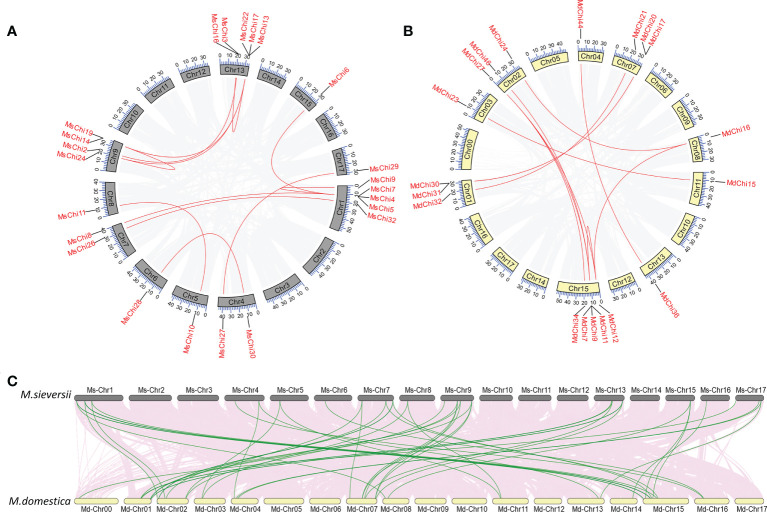
Collinearity analysis of *MsChis* and *MdChis*. Distribution of segmental duplication of chitinase genes on *M.sieversii* **(A)** and *M.domestica* **(B)** chromosomes. Chromosomes are represented by gray or yellow boxes. Segmental duplication genes are connected with red lines. The gray lines indicate all synteny blocks within the genome, and the chromosome numbers were indicated at the inside of the box. **(C)** Synteny analysis of chitinase genes in *M.sieversii* and *M.domestica*. Gray lines in the background indicate the collinear blocks within *M.sieversii* and *M.domestica* genomes, while the red lines highlight the syntenic chitinase gene pairs.

In the course of evolution, gene duplication plays a crucial role in the expansion of a gene family (Cannon et al., 2004; Yanai, 2022). To further study the expansion mechanism of the chitinase genes, we employed the basic local alignment search tool for proteins (BLASTP) and MCScanX tools to identify duplication events in the chitinase gene family of the two apple species. Segmentally duplicated genes in the *M. sieversii* and *M.* × *domestica* genomes were identified by collinearity analysis (**Figures 2A, B**). In detail, 14 pairs of *MsChi* genes were segmentally duplicated among 10 chromosomes, whereas 12 *MdChi* genes were segmentally duplicated among nine chromosomes (**Table S4**). In addition, 16 and 22 chitinase genes were tandemly duplicated in *M. sieversii* and *M.* × *domestica*, respectively (**Table S4**). These results demonstrated that tandem duplication was a major driving force in expansion of the chitinase gene family in *M. sieversii* and *M.* × *domestica*.

To further explore the evolutionary history of the chitinase family, we calculated the *K*_a_ and *K*_s_ substitution rates for duplicated chitinase gene pairs (**Table S4**). Almost half of the segmental or tandem duplicated *MsChi* genes were subjected to positive selection (*K*_a_/*K*_s_ > 1), whereas the remainder had undergone purifying selection (*K*_a_/*K*_s_ < 1). The segmental or tandem duplication of *MsChi* genes was estimated to have occurred from 3.62 to 128.71 Mya. Among *MdChi* genes, purifying selection was dominant for segmental duplication, whereas positive selection accounted for the highest proportion of tandem duplication events. Duplication of *MdChi* genes was estimated to have occurred between 1.93 and 138.52 Mya.

To understand the orthologous relationships within the *M. sieversii* and *M.* × *domestica*, we further analyzed the synteny of chitinase genes. A tightly conserved collinearity relationship and strong orthologs of chitinase genes were observed between the syntenic regions among the *M. sieversii* and *M.* × *domestica* genomes (**Figure 2C**). Thirty orthologous chitinase gene pairs were identified between *M. sieversii* and *M.* × *domestica* (**Table S5**).

### Gene structure and conservative motif analyses

**Figure 3:**
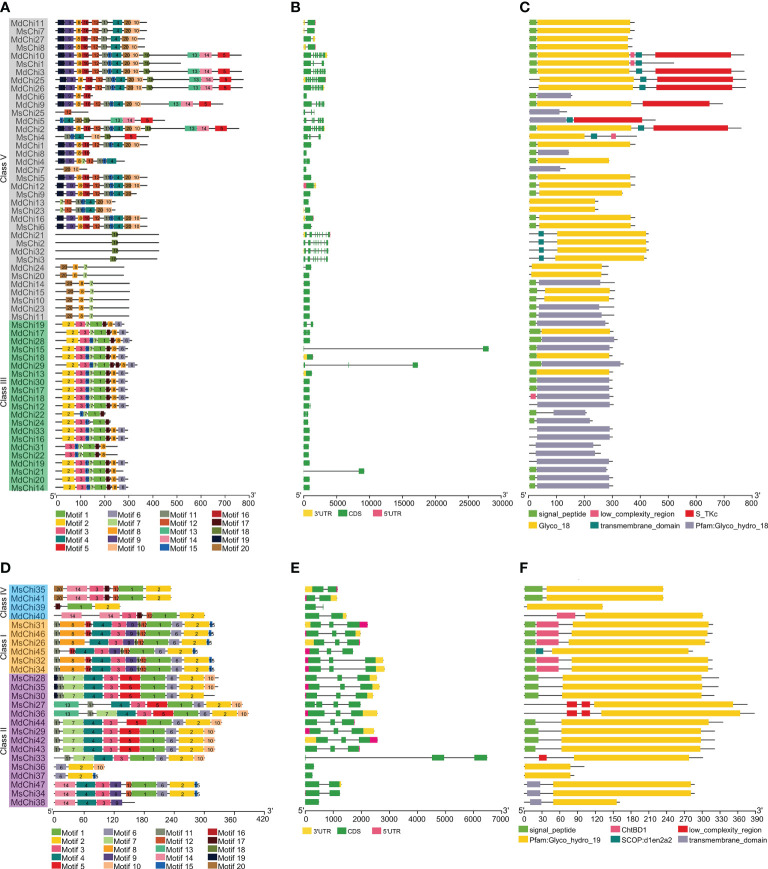
Structural analysis *MsChis* and *MdChis*. The conserved motifs in GH18 chitinase subfamily **(A)** and GH19 chitinase **(D)** proteins from *M.domestica* and *M.sieversii* were identified by MEME (Bailey et al., 2006). Schematic representation of exon-intron structure of GH18 **(B)** and GH19 **(E)** chitinase subfamilies in *M.domestica* and *M.sieversii*. Active domains of GH18 **(C)** and GH19 **(F)** chitinase proteins from *M.sieversii* and *M.domestica* were identified by SMART (Letunic et al., 2021) database. Motifs, domains and exon-intron structures were visualized by TBtools software (Chen et al., 2020). Each single signature is indicated by a colored box on bottoms of the figure and presented proportionally.

To determine structural features of the chitinase genes from the two apple species, we analyzed the exon–intron distribution of the genes. Most GH18 chitinase genes contained one or two exons, except for the genes of Class V, which contained seven or eight exons (**Figure 3B**). In contrast, three exons were most common for GH19 chitinase genes (**Figure 3E**). Interestingly, three genes (*MsChi15*, *MsChi21*, and *MdChi29*) in Class III (**Figure 3B**) and only one gene (*MsChi33*) in Class II contained relatively long introns (**Figure 3E**). Most of the closely related genes in the two apple species were similar in length and number of exons/introns.

To further study the architecture of chitinase proteins, the conserved motifs were predicted with the MEME Suite. A total of 20 distinct motifs were identified (**Figures 3A, D**). The number and location of motifs of certain closely related genes in the two apple species were highly conserved, providing further evidence to support the phylogenetic and functional relationships. Furthermore, several class-specific motifs were detected in the GH18 family. For instance, motifs1, 2, and 17 were restricted to Class III, wheras motif18 was the only motif in some members of Class V (**Figure 3A**). Motif2 was common in each class of GH19, except in MdChi38. In addition, motif17 and motif16 were unique to Class IV and Class I, respectively (**Figure 3D**).

### Conserved domains and active site analysis of chitinase genes

**Figure 4:**
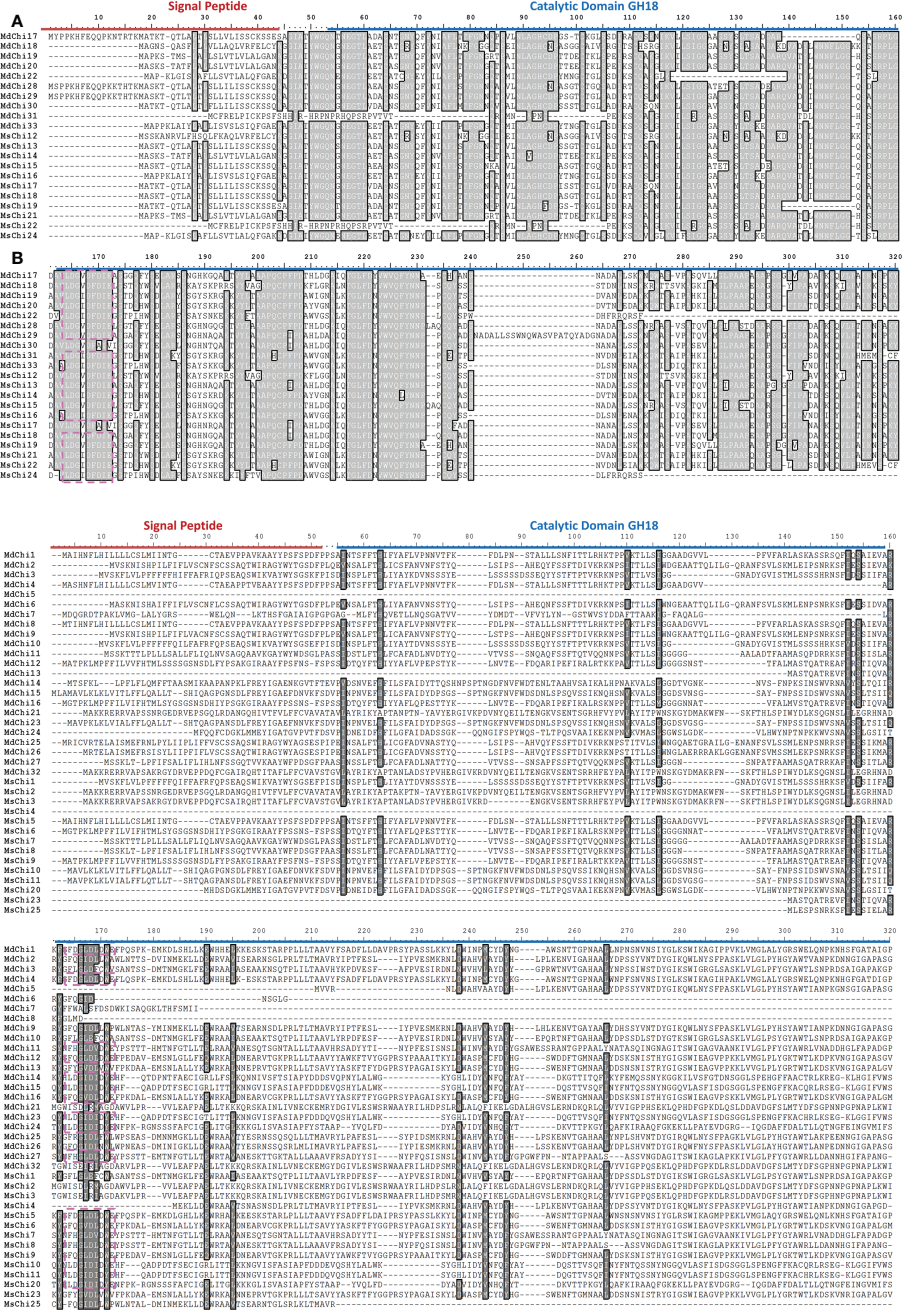
Multiple alignments of GH18 chitinase subfamily Class III **(A)** and Class V **(B)** of *MsChis* and *MdChis*. Amino acid sequences were aligned using ClustalW and visualized using BioEdit. Shaded amino acid sequences are 75–100% homologous. Red line indicates signal peptide sequence and blue line for GH18 catalytic domain. The amino acids in the purple dash line box represent residues essential for catalytic activity, Chitinase_18 signature (PS01095). The partial amnio acid sequences were presented in **(B)**.

**Figure 5:**
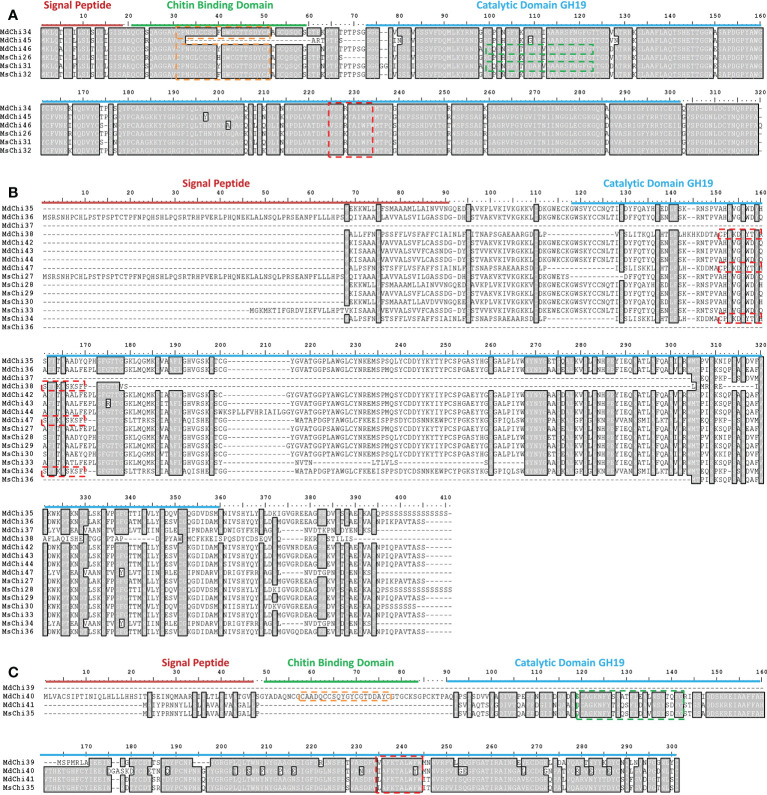
Multiple alignments of GH19 chitinase subfamily Class I **(A)**, Class II **(B)** and Class IV **(C)** of *MsChis* and *MdChis*. Amino acid sequences were aligned using Clustal W and visualized by using BioEdit. Shaded amino acid sequences are 75–100% homologous. Red line indicates signal sequence and blue line for GH19 catalytic domain. The amino acids in the green dash line box represents CHITINASE_19 _1 signature (PS00773); red dash line box represents CHITINASE_19_2 signature (PS00774); orange dash line box represents CHIT_BIND_I_1 signature (PS00026).

To examine the similarity and diversity of domain architecture of chitinase proteins from the two apple species, the localization of the catalytic domain within the protein was analyzed by searching for featured domains using the SMART database. Glycosyl hydrolase family 18 (GH18) domains were present in classes III and V chitinases, and Glycosyl hydrolase family 19 (GH19) domains were detected in classes I, II, and IV. To locate the catalytic domain and active site in each chitinase protein, we generated a multiple sequence alignment and conducted a motif-based sequence analysis. Analysis of the amino acid sequences combined with the domain signature revealed that Class III chitinases possessed the GH18 catalytic domain with the CHITINASE_18 active site signature (PS01095, [LIVMFY]-[DN]-G-[LIVMF]-[DN]-[LIVMF]-[DN]-x-E) except for MdChi30 and MdChi17 (**Figure 4A**). Among Class V chitinases, 10 members had the CHITINASE_18 active site signature (**Figure 4B**). The Class I chitinases contained the GH19 catalytic domain with the CHITINASE_19_2 signature (PS00774; [LIVM]-[GSA]-F-x-[STAG](2)-[LIVMFY]-W-[FY]-W-[LIVM]) and CHITINASE_19_1 signature (PS00773; C-x(4,5)-F-Y-[ST]-x(3)-[FY]-[LIVMF]-x-A-x(3)-[YF]-x(2)-F-[GSA]) (**Figure 5A**). In addition, Class I chitinases had the chitin-binding domain with the CHIT_BIND_I_1 signature (PS00026; C-x(4,5)-C-C-S-x(2)-G-x-C-G-x(3,4)-[FYW]-C) except for MdChi45. Class IV chitinases contained the GH19 catalytic domain with the CHITINASE_19_1 and CHITINASE_19_2 signatures. Only MdChi40 had the chitin-binding domain with the CHIT_BIND_I_1 signature (**Figure 5B**). Among Class II chitinases, the CHITINASE_19_1 signature was detected in only three members (MdChi38, MdChi47, and MsChi34), and no CHITINASE_19_2 signature was detected in any members of this class (**Figure 5C**).

### Prediction of potential *cis*-acting elements in chitinase genes

To explore the transcriptional modulation of the chitinase genes, we predicted the presence of *cis*-acting elements in the promoter region (within 2000 bp upstream of the coding region) of the genes in the two apple genomes. The number of *cis*-acting elements involved in response to drought, low temperature, light, and phytohormones were predicted (**Figures S1**, **S2**). Among these *cis*-acting elements, the W-box (TTGACC) (Rushton and Somssich, 1998), P-box (CCTTTTG) (Lois et al., 1989), L-box (ATCCCACCTAC) (Lois et al., 1989), and S-box (AGCCACC) (Kirsch et al., 2000) are reported to respond to fungal pathogen infection. The most frequent *cis*-acting elements in all promoters were the TATA-box, CAAT-box, and AT-TATA box.

### Expression of chitinase genes in response to *V. mali* inoculation

**Figure 6:**
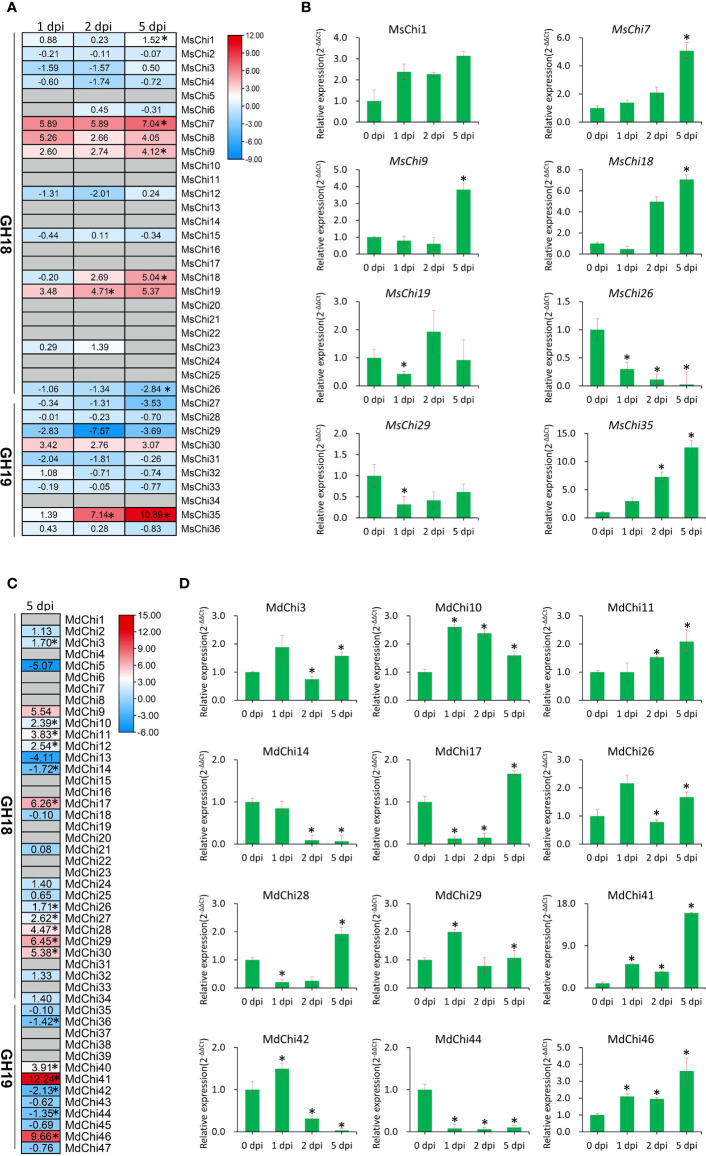
Expression profile of *MsChis* and *MdChis* during *V.mali* infection. RNA-seq data analysis of chitinase genes in *M.sieversii* **(A)** or *M.domestica* **(C)** in response to *V.mali*. The heatmap generated by using TBtools (Chen et al., 2020) software based on the log2fold change values of each chitinase genes. The colors represent expression levels, red colors represent high expression and blue colors represent low expression. ‘*’ represented significantly (*P*<0.05) different expression. Verification of the expression of chitinase genes in *M.sieversii* **(B)** or *M.domestica* **(D)** under *V.mali* infection by qRT-PCR. *EF1-α* was used as an internal reference gene. The relative expression data were analyzed by one-way ANOVA method. ‘*’ represented significantly different (*P*< 0.05, n = 3), and error bars indicate mean ± SE.

To investigate expression of the chitinase genes of *M. sieversii* and *M. domestica* in response to *V. mali* attack, we analyzed the transcriptome of *M. sieversii* and *M.* domestica leaves after inoculation with *V. mali*. A total of 36 MsChi transcripts from *M. sieversii* were subjected to further differential expression analysis through comparisons of fold-change expression. Five *M. sieversii* GH18 genes (*MsChi1*, *MsChi7*, *MsChi9*, *MsChi18*, and *MsChi19*) and one *M.* domestica GH19 gene (*MsChi35*) were up-regulated, whereas a single *M. sieversii* GH18 gene (*MsChi26*) was down-regulated significantly in response to *V. mali* infection (**Figure 6A**). For *M. domestica*, we analyzed previous transcriptome data in response to *V. mali* infection (NCBI SRA accession ID : SRP034726) (Yin et al., 2016). Thirteen genes were up-regulated, whereas four genes were down-regulated (**Figure 6C**). To validate the expression pattern of each transcript, we quantified the transcript abundance of each *MsChi* and *MdChi* gene by qRT-PCR at different time points after inoculation. The qRT-PCR results for the *MsChi* transcripts were consistent with the RNA-sequencing data (**Figures 6B, D**).

### Co-expression network of *MsChi* genes

**Figure 7:**
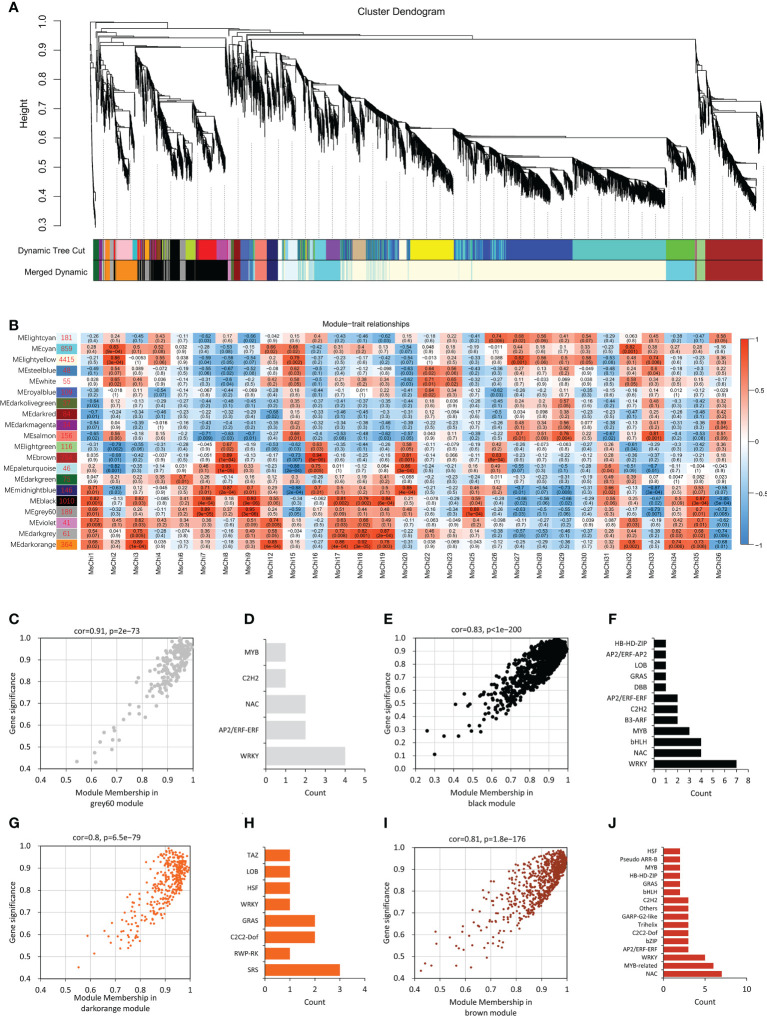
Weighted Gene Co-Expression Network Analysis (WGCNA) of *MsChi* genes. **(A)** Gene dendrogram obtained using dynamic tree cut-off. Each colored row in the bottom represents a module which contains group of highly connected genes. **(B)** Association of module eigengenes (MEs) with expression profiles of selected chitinase genes. Corresponding *P*-values of module-chitinase gene expression correlations are indicated in parenthesis. The panel on the left side shows the 20 modules and number of genes in each module. The color scale on the right side shows module-trait correlation from −1 (blue) to 1 (red). Scatter plot of the correlation of module membership vs. gene significance in grey60 **(C)**, black **(E)**, darkorange **(G)** and brown modules **(I)**. Statistics of transcription factors in grey60 **(D)**, black **(F)**, orange **(H)** and brown modules **(J)**.

To further predict the transcription factors associated with chitinase gene expression, we preformed WGCNA based on the FPKM values from the RNA-sequencing data. After filtering (FPKM > 1), 8780 genes were subjected to further analysis and 20 co-expressed gene modules were identified. A module with a correlation coefficient (*r*) > 0.80 and *p* < 0.05 was defined as a *MsChi*-specific module. In this manner, 10 *MsChi*-specific modules were identified (**Figure 7B**). Notably, the MEblack module with 1010 genes was positively correlated with six up-regulated *MsChi* genes (*MsChi1*, *MsChi7*, *MsChi9*, *MsChi18*, *MsChi19*, and *MsChi35*) in response to *V. mali* infection. Calculation of MM and GS for the 1010 genes in the MEblack module revealed that 55.6% of the genes (562/1010) had high MM (>0.8) and GS (>0.8) scores, implying that they had relatively high connectivity (*r* = 0.83, *p* < 1e−200) within the module (**Figure 7C**). Twenty-nine genes were identified as transcription factors in the MEblack module of which seven were WRKY transcription factors (**Figure 7D**). These results inferred that WRKY transcription factors may function as upstream positive regulators of a majority of the *MsChi* genes in response to *V. mali* infection.

## Discussion

Apple is among the most commonly consumed fruits worldwide. Fungal diseases, such as Valsa canker, are a major threat to apple production. Fungal resistance proteins, such as chitinases, are essential to prevent canker diseases in apple. Chitinases are synthesized in diverse organisms, including bacteria, fungi, plants, insects, animals, and humans (Hamid et al., 2013). The chitinase gene family has been identified in numerous plant species, such as vegetable (Bartholomew et al., [[NoYear]]; Cao and Tan, 2019; Mir et al., 2020), crop (Su et al., 2015; Xu et al., 2016), and tree species (Tobias et al., 2017; Zhang et al., 2022). Reliable genomic sequence information and annotations are available for a number of *Malus* species (Daccord et al., 2017). However, genome-wide identification of the chitinase gene family and its expression patterns under fungal infection have not been reported previously for apple species. In the present study, 36 and 47 putative chitinase genes were identified in the genomes of the wild apple *M. sieversii* and domesticated apple *M. domestica*, respectively. Based on their phylogenetic relationships and functional domains, the chitinases were classified into five classes (I–V).

It is noteworthy that the chitinase genes were not evenly distributed on chromosomes. The chitinase genes of wild apple were mainly concentrated on chromosomes 1, 7, 9, 13, and 17, whereas those of domesticated apple were concentrated on chromosomes 1, 2, 4, 7, and 15 (**Figures 1A, B**). In some instances, the chitinase genes clustered on individual chromosomes were members of the same class. For instance, *MdChi2*, *MdChi5*, *MdChi9*, and *MdChi10* belonged to class V and were clustered on chromosome 15 (**Table S4**). The distribution pattern of chitinases in wild and domesticated apple showed similarity with the genome of other tree species, such as *E. grandis* (Tobias et al., 2017).

Human activity, including agricultural breeding and genetic engineering, have driven rapid evolutionary changes in domesticated plants and animals (Palumbi, 2001; Turcotte et al., 2017). We identified a greater number of chitinase genes in domesticated apple compared with wild apple, reflecting that domestication drives evolution. The structural diversity of chitinase genes may provide an improved understanding of the evolutionary patterns of chitinase genes. The average exon number of each class of chitinase genes has remained almost unchanged in wild and domesticated apple, and Class V chitinase genes have more exons compare with the other classes (**Figure S3**). For instance, the exon number of 28 *MdChi* genes preserved the number in ancestral orthologs (**Table S7**), implying that the gene structure has remained conserved during evolution. With regard to intron number, stress-responsive genes generally contain relatively fewer introns (Jeffares et al., 2008). For example, the cotton Class IV chitinase gene *GrChi28* has a single intron and is strongly up-regulated in response to *Verticillium dahliae* infection (Xu et al., 2016). Similarly, *MsChi35* (Class IV) and *MdChi41* (Class IV) were strongly up-regulated (**Figure 6**) after *V. mali* inoculation and each contained a single intron (**Figure 3D**). In accordance with this pattern, all significantly up- or down-regulated chitinase genes in wild or domesticated apple contained two or fewer introns (**Figure 3**).

Gene duplication is a major driving force of expansion of a gene family. The expansion of chitinase genes in wild and domesticated apple resulted from segmental duplication and tandem duplication. Duplicated genes likely experience functional specification (Prince and Pickett, 2002). In wild apple, 27% of chitinase genes originated from tandem duplication, whereas 36% were segmentally duplicated (**Table S4**). These duplicated chitinase genes maintained a close phylogenetic relationship but structural variation was evident. For instance, *MsChi25* lost the signal peptide sequence compared with its paralog *MsChi8*. The genes *MsChi1*, *MsChi4*, and *MsChi7* formed a tandemly arrayed gene cluster, but only *MsChi7* possessed the CHITINASE_18 active site signature (PS01095) in the GH18 catalytic domain (**Figure 4B**) and was strongly up-regulated under infection by *V. mali*. In addition, *MsChi13*, *MsChi15*, *MsChi17* and *MsChi18* were tandemly arrayed on chromosome 13, but exhibited various expression patterns in response to *V. mali* infection. Similarly, duplicated chitinase genes in domesticated apple showed high structural variety despite their phylogenetic affinity. For example, *MdChi40* and *MdChi41* were tandemly duplicated gene pairs under purifying selection, but MdChi40 gained a single chitin-binding domain at the N-terminus (**Figure 5C**). Interestingly, *MdChi41* was up-regulated in response to *V. mali* infection and its expression level was ten-times higher than that of *MdChi40*. We hypothesize that the gain of the chitin-binding region of *MdChi40* may affect its enzymatic activity and lead to functional diversification. Although the present results infer that duplicated genes may acquire a new structure and function, additional evidence is required to confirm the functional differences between duplicated chitinase genes.

Chitinases vary in molecular structure, substrate specificity, and catalytic mechanism (Hamid et al., 2013). In the current study, structural variation of the chitinase proteins was detected. Similar motif composition was observed in classes I and III, suggesting that these classes show functional similarity (**Figure 3**). The variation in motif composition was not obvious between classes II and IV, but very distinct in Class V chitinases (**Figure 3**). In addition, a majority of the chitinase proteins in this study included a signal peptide, which was absent in several chitinase proteins. Plant chitinase proteins mature by trimming of the N-terminal signal peptide (Taira et al., 2009) and confer enhanced plant resistance to fungal pathogens (Singh et al., 2015). Chitinase genes that were significantly up-regulated in response to *V. mali* infection all carried the signal peptide sequence, indicating that they are secreted into the apoplast and may be involved in plant–pathogen interaction.

To explore the mechanism by which the chitinase genes were transcriptionally regulated, we analyzed the promoter regions of the chitinase genes. We detected a number of pathogen-responsive *cis*-acting elements, such as the W-box. Some of the chitinase genes, such as *MsChi35* or its ortholog *MdChi41*, were observed to have two W-box *cis*-elements in the promoter region (**Figures S1**, **S2**) and were strongly up-regulated in response to *V. mali* infection (**Figure 6**). These results indicated that expression of *MsChi35* and *MdChi41* was likely regulated *via* the W-box *cis*-acting element in the promoter region. A gene co-expression network revealed that *MsChi35* was highly co-expressed with seven WRKY transcription factors under *V. mali* infection (**Figure 7**). Further analysis showed that six of the seven WRKY transcription factors were significantly differentially expressed at 5 dpi (**Figure S4**). WRKY transcription factors are among the largest families of transcriptional regulators in plants and bind to the W-box *cis*-acting element in the promoter of the target gene (Zhu et al., 2017). In *Lilium regale*, LrWRKY2 can activate expression of *LrCHI2*, which encodes a chitinase, through binding to the W-box *cis*-element in the promoter of *LrCHI2* to enhance host resistance to root rot caused by *Fusarium oxysporum* (Li et al., 2021). The tobacco Class I chitinase gene *NtCHN48* has two W-boxes in the promoter region that are recognized by NtWRKY1 and NtWRKY4, respectively (Yamamoto et al., 2004). In a previous study, we demonstrated that MsWRKY16, one of the seven WRKY transcription factors, enhances resistance to *V. mali* (Liu et al., 2021). Based on these results, it is speculated that WRKY transcription factors bind to the W-box *cis*-element in the promoter region of *MsChi35* or *MdChi41* to positively regulate their expression in response to *V. mali* infection. The genes *MsChi35* and *MdChi41* are orthologs belonging to Class IV chitinase. The two genes were highly conserved in their composition and arrangement of exons–introns (**Figure 3E**) or motifs (**Figure 3D**) and shared a high level of homology (100%) in amino acid sequence and enzymatic domain (**Figure 5C**). The *MsChi35* and *MdChi41* were the most highly expressed chitinase genes in response to *V. mali* infection. Moreover, the transient over expression of *MsChi35* enhance *M.sieverssi* resistance to *V. mali* infection (**Figure S5**). Therefore, these results suggested that *MsChi35* and *MdChi41* were the dominant fungal resistance chitinase genes in the two apple species.

**Figure 8:**
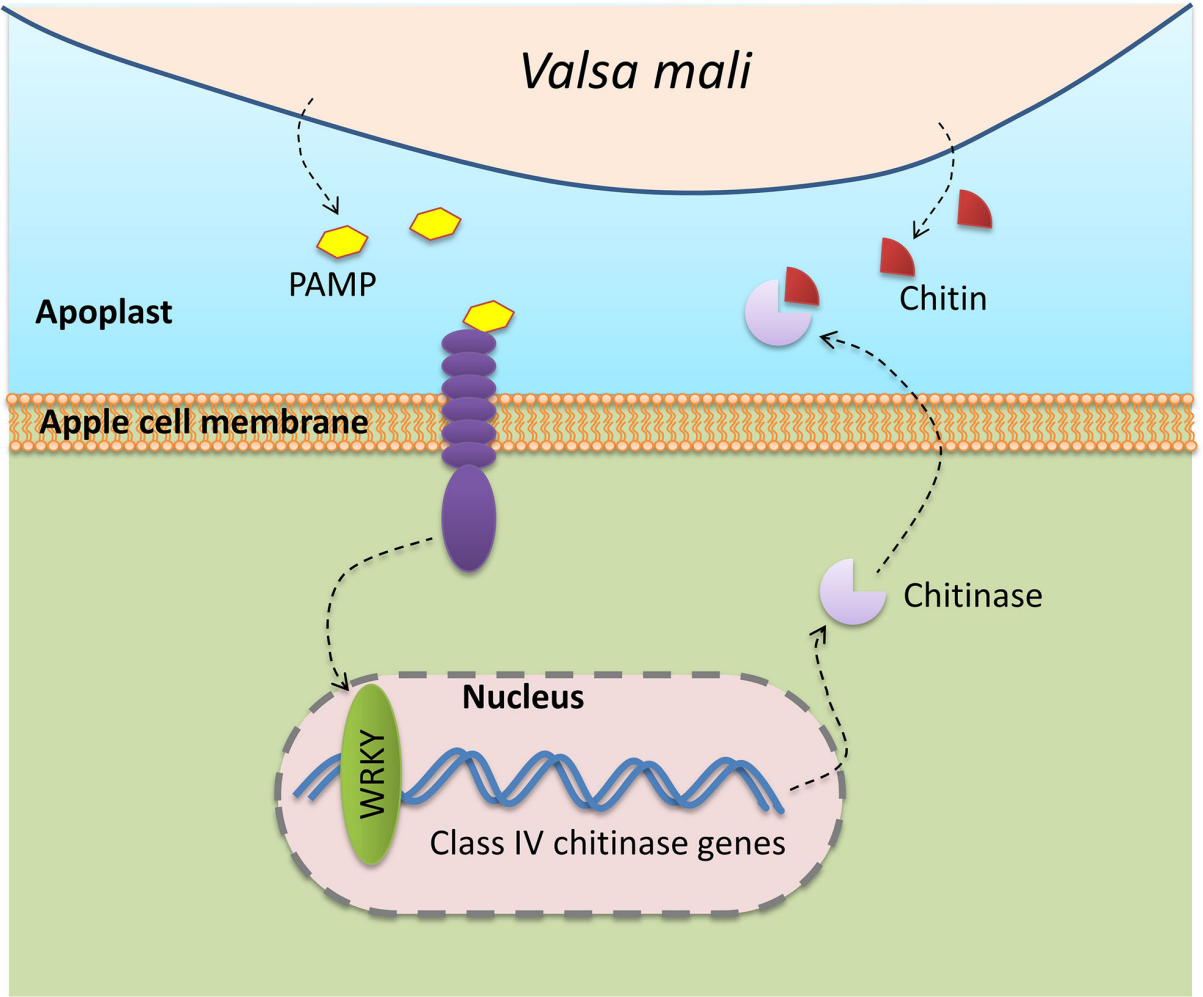
Schematic model illustrating the responses of apple chitinase family genes against to *V.mali* infection.

The present research allows proposal of a framework for the response of chitinase family genes to *V. mali* infection in *M. sieversii* and *M. domestica* (**Figure 8**). The results provide useful insights for further functional investigation of the chitinase gene family, particularly of *MsChi35* and *MdChi41*, in the mediation of fungal pathogen resistance in apple species.

## Data availability statement

Publicly available datasets were analyzed in this study. This data can be found here: The *M.sieversii* transcriptome datasets that used in the current article are available at the Sequence Read Archive (SRA) database of National Center for Biotechnology Information under project accession number PRJNA687214(https://www.ncbi.nlm.nih.gov/bioproject/?term=PRJNA687214). The *M.domestica* transcriptom datasets that used in the current article are available at the Sequence Read Archive (SRA) database of National Center for Biotechnology Information under accession number SRP034726 (https://www.ncbi.nlm.nih.gov/sra/?term=SRP034726). The *M.sieversii* genome data are available at genome database of National Center for Biotechnology Information under accession number JAHTLV000000000.1 (https://www.ncbi.nlm.nih.gov/nuccore/JAHTLV000000000.1). The *M.domestica* genome data are available at https://iris.angers.inra.fr/gddh13/the-apple-genome-downloads.html.

## Author contributions

The experimental design, data analyzation, manuscript organization were completed by YH and GK. XZ, XL (6^th^ author) and XW were assistant with RNA quantification. DZ and XL (8^th^ author) conceived the project, supervised the analysis, and critically revised the manuscript. AW was revised the manuscript. All authors have read and agreed to the published version of the manuscript.

## Funding

This work was funded by the Second Tibetan Plateau Scientific Expedition and Research (STEP) program (2019QZKK0502030403), Biological Resources Program, Chinese Academy of Sciences (KFJ-BRP-007-008) and the Youth Innovation Promotion Association, Chinese Academy of Sciences (No. 2018478) and the China Postdoctoral Science Foundation (2021M693380).

## Conflict of interest

The authors declare that the research was conducted in the absence of any commercial or financial relationships that could be construed as a potential conflict of interest.

## Publisher’s note

All claims expressed in this article are solely those of the authors and do not necessarily represent those of their affiliated organizations, or those of the publisher, the editors and the reviewers. Any product that may be evaluated in this article, or claim that may be made by its manufacturer, is not guaranteed or endorsed by the publisher.

## Supplementary material

Supplementary Table S1 List of the qRT-PCR primers of the MsChi and MdChi genes.

Supplementary Table S2 Detailed information of predicted chitinase genes in *M.sieversii* and *M.domestica*.

Supplementary Table S3 Number of different classes of chitinase from different species.

Supplementary Table S4 Duplicated chitinase genes in *M.sieversii* and *M.domestica*.

Supplementary Table S5 Ortholog chitinase genes in *M.sieversii* and *M.domestica*.

Supplementary Table S6 RNA-seq expression data of seven WRKY transcription factors in *M.sieversii* in respons to V.mali infection.

Supplementary Table S7 Exon number in the ortholog pairs of MsChi and MdChi genes.
