# Supplementary figures and images for: Genome-wide characterization of the chitinase gene family in wild apple (Malus sieversii) and domesticated apple (Malus domestica) reveals its role in resistance to Valsa mali
Source: Front Plant Sci. 2022 Nov 7;13:1007936. doi: 10.3389/fpls.2022.1007936 (PMC9676469; doi:10.3389/fpls.2022.1007936)

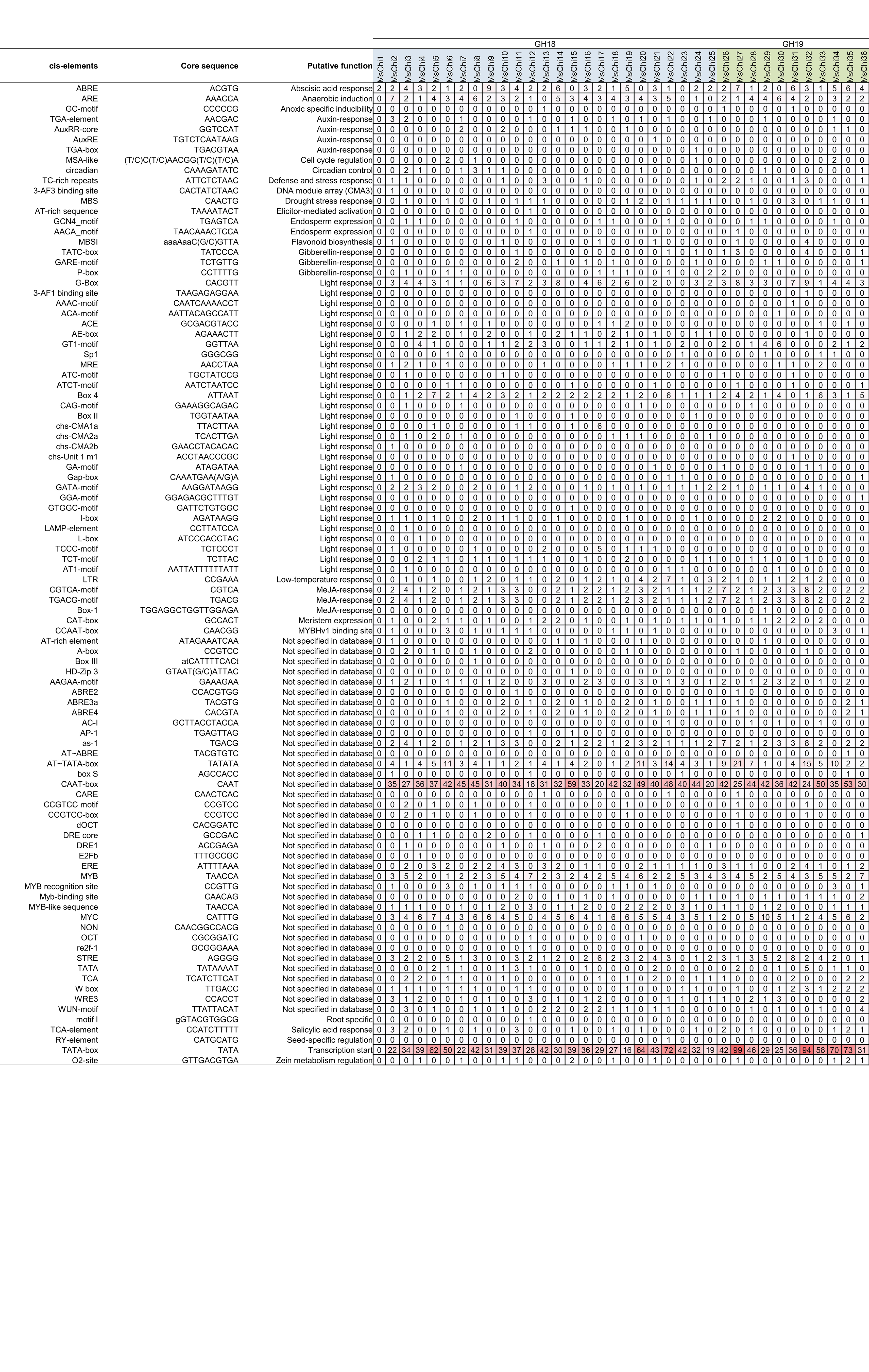

Supplement: Supplementary file 2 [file Image_1.jpeg]

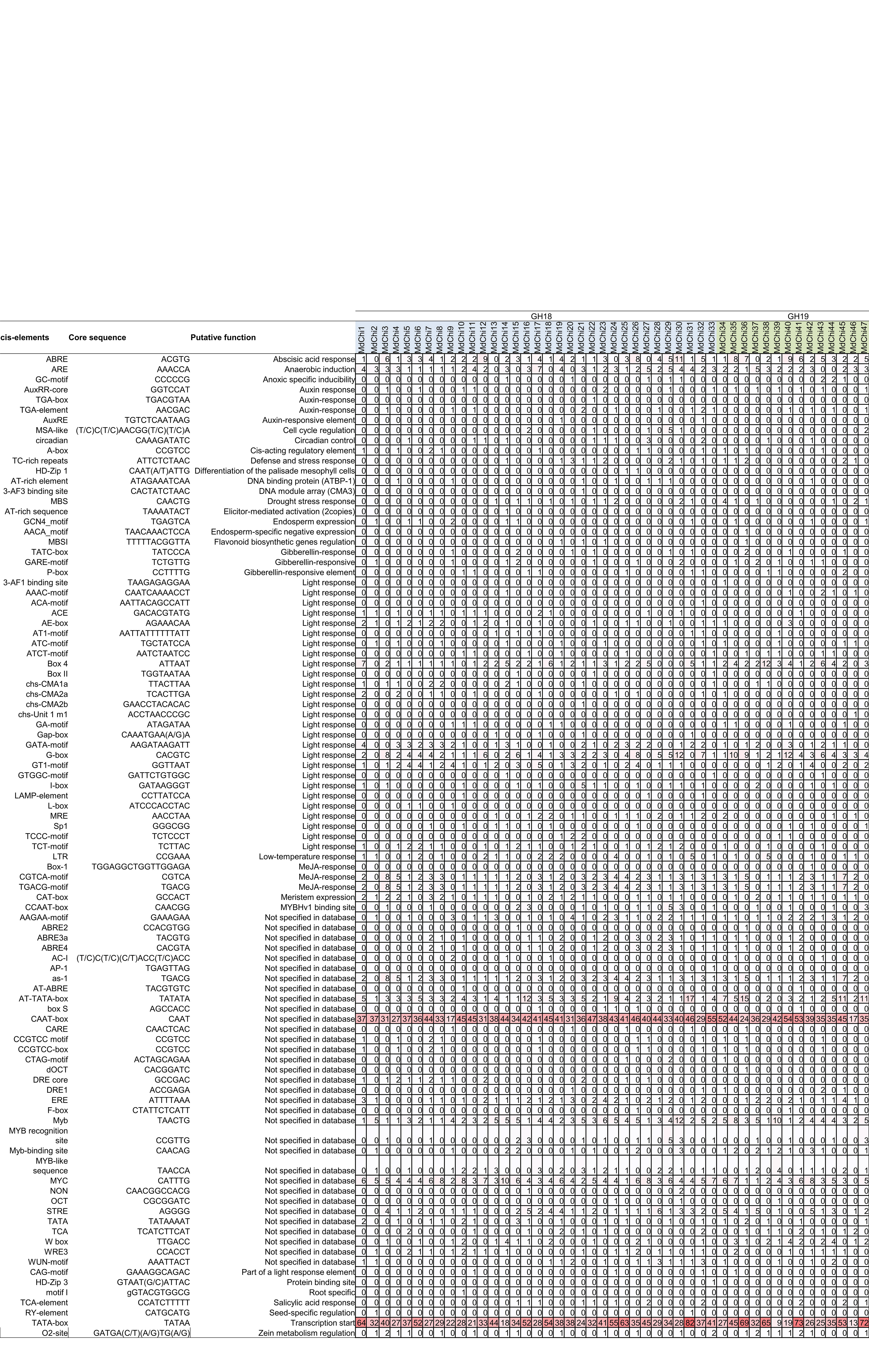

Supplement: Supplementary file 3 [file Image_2.jpeg]

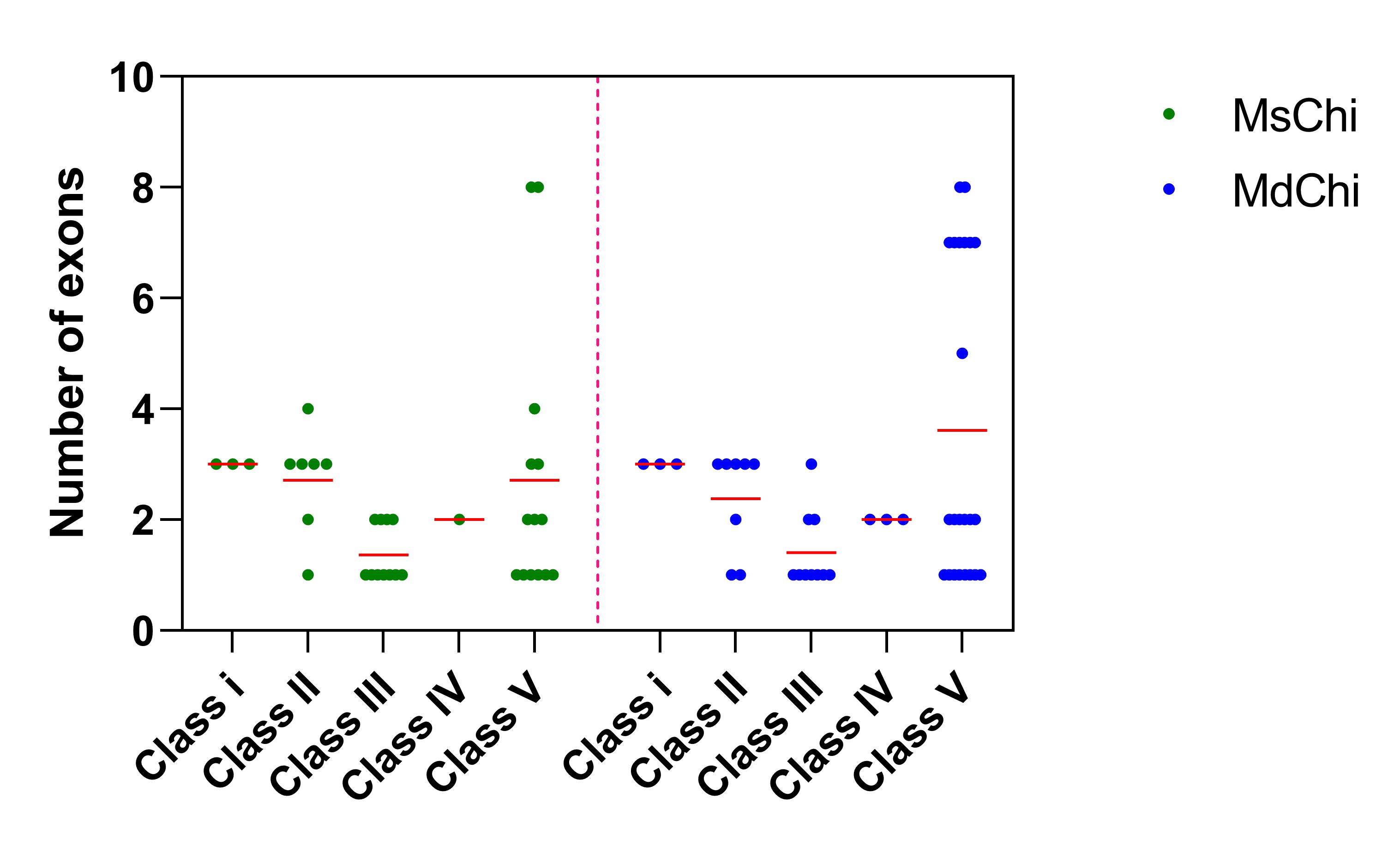

Supplement: Supplementary file 4 [file Image_3.jpeg]
